# Comparisons of cancer detection rate and complications between transrectal and transperineal prostate biopsy approaches - a single center preliminary study

**DOI:** 10.1186/s12894-019-0539-4

**Published:** 2019-10-28

**Authors:** Guan-Lin Huang, Chih-Hsiung Kang, Wei-Ching Lee, Po-Hui Chiang

**Affiliations:** Department of Urology, Kaohsiung Chang Gung Memorial Hospital and Chang Gung University College of Medicine, No.123, Dapi Rd., Niaosong Dist., Kaohsiung, Taiwan, Republic of China

**Keywords:** Prostate biopsy, Transperineal (TP), Transrectal (TR), Local anesthesia

## Abstract

**Background:**

Prostate biopsy remains the gold standard approach to verify prostate cancer diagnosis. Transrectal (TR) biopsy is a regular modality, while transperineal (TP) biopsy is an alternative for the patients who display persistently high levels of prostate-specific antigen (PSA) and thus have to undergo repeat biopsy. This study aimed to compare the cancer detection rates between TR and TP approaches and assess the post-bioptic complications of the two procedures. Besides, the feasibility of performing TP biopsies under local anesthesia was also evaluated.

**Methods:**

A total of 238 outpatient visits meeting the criteria for prostate cancer biopsy were enrolled for this study. They were divided into two groups: the TP group (*n* = 130) consists of patients destined to undergo local anesthetic TP biopsy; and the TR group (*n* = 108) contained those who received TR biopsy as comparison. Age, PSA level, digital rectal exam (DRE) finding, prostate volume, and biopsy core number were used as the parameters of the multivariable analyses. The comparable items included cancer detection rate, complication rate, admission rate and visual analog scale (VAS) score.

**Results:**

The cancer detection rates between TP and TR groups were quite comparable (45% v.s. 49%) (*p* = 0.492). However, the TP group, as compared to the TR group, had significantly lower incidence of infection-related complications (except epididymitis and prostatitis) that commonly occur after biopsies. None of the patients in the TP group were hospitalized due to the post-bioptic complications, whereas there was still a minor portion of those in the TR group (7.4%) requiring hospitalization after biopsy. Medians (25–75% quartiles) of visual analog scale (VAS) were 3 [3, 4] and 4 [3–5] respectively for the TP and TR procedures under local anesthesia, but no statistical significance existed between them (*p = 0.085*).

**Conclusions:**

Patients receiving TP biopsy are less likely to manifest infection-related complications. Therefore, TP biopsy is a more feasible local anesthetic approach for prostate cancer detection if there are concerns for infectious complications and/or the risk of general anesthesia.

## Background

The transrectal (TR) ultrasound-guided biopsy is the gold standard approach for prostate cancer diagnosis [1, 2]. Though being generally considered a relatively low-risk outpatient approach, up to 50% of the patients suffer from minor complications (e.g. hematuria, hematospermia, rectal bleeding, and acute urine retention) to severe complications (e.g. anemia and syncope) [3]. Approximately 4 to 5% of the patients who undergo this procedure require hospital admission due to infection-related complications (ranging from bacteriuria to sepsis) [4, 5]. Therefore, prostate biopsy performed through other approaches should be considered.

Transperineal (TP) biopsy is an alternative approach for patients required to undergo prostate repeat biopsy. This procedure has been shown to greatly improve the cancer detection rates in the anterior and apical areas of the prostate and also reduce the risk for infectious complications [6, 7]. Nevertheless, TP biopsy is not used as widely as TR biopsy owing to the relatively higher technical difficulty and the pain occurring without the use of anesthetics. In this study, we compared the rates of cancer detection and post-bioptic complications between the Taiwanese patients receiving transperineal (TP) and transrectal (TR) prostate biopsies, respectively. Comparisons of visual analog scale (VAS) scores between these two patient groups were done for evaluating the feasibility of TP under local anesthesia.

## Methods

### Patients

Three hundred and fifty-six patients were recruited to receive the prostate biopsy at our institute from May 2015 to Dec 2017. All patients enrolled in this IRB (IRB number:105-6117B)-approved study were given the informed consent. Biopsy criteria included elevated PSA level above 4.0 ng/mL, abnormal digital rectal examination findings, and clinical suspicion of prostate cancer. Exclusion criteria of the patients were listed as follows: (1) having previously undergone prostate biopsy, (2) clinically having locally advanced prostate cancer (verified by DRE) with significant bone pain or bone metastasis, (3) showing pathological evidence of metastatic prostate cancer. After excluding the non-qualifiers, 130 and 108 patients were respectively assigned to the TP and TR groups by their attending physicians (TP group: PHC; TR group: CSK & WCL). The patients were clearly and concisely informed of the benefits and the risk separately from the TP and TR bioptic procedures via an oral explanation, and the letter of their consent was acquired. Digital rectal examination, negative urinalysis, and PSA evaluation were carried out in all male patients prior to biopsy.

### Biopsy protocols

For transrectal (TR) prostate biopsy: patients received the perioperative oral antibiotics (empirically using levofloxacin/ceftibuten/baktar, or others according to the culture data) for 3 days with pre-procedure 1pc Gentamicin IM injection. Biopsy gun (BARD MAXCORE/18*20; Bard, Tempe, USA) was applied through the transrectal ultrasonography (bk3000; BK Medical Aps, Copenhagen, Denmark) with full lidocaine cream lubrication.

**Fig. 1:**
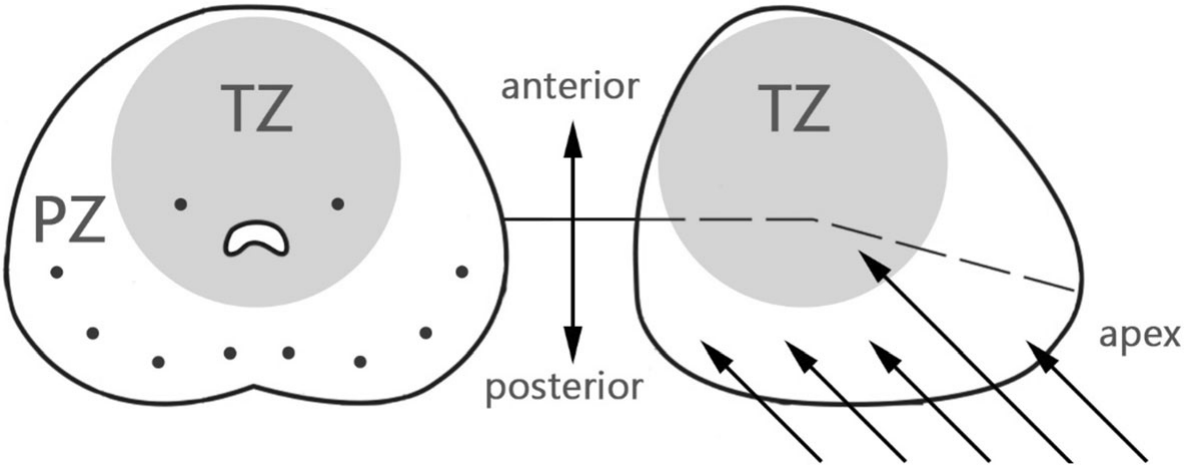
Transrectal prostate biopsy mapping. A total of 10 pieces of biopsy tissue including 2 pieces focusing on transitional zone were taken. Large prostate or significant prostate lesion under transrectal ultrasound may need additional pieces by clinical judgement. TZ: transitional zone. PZ: peripheral zone

Generally, the 10 core biopsy included 8 slices from the apex-mid plane-basal peripheral zone (PZ) containing the far lateral area as well as 2 slices from the bilateral transitional zone (TZ) via the probe guided-side hole. (Fig. 1.) The biopsy number might vary according to the prostate volume or additional suspicious transrectal ultrasound findings.

**Fig. 2:**
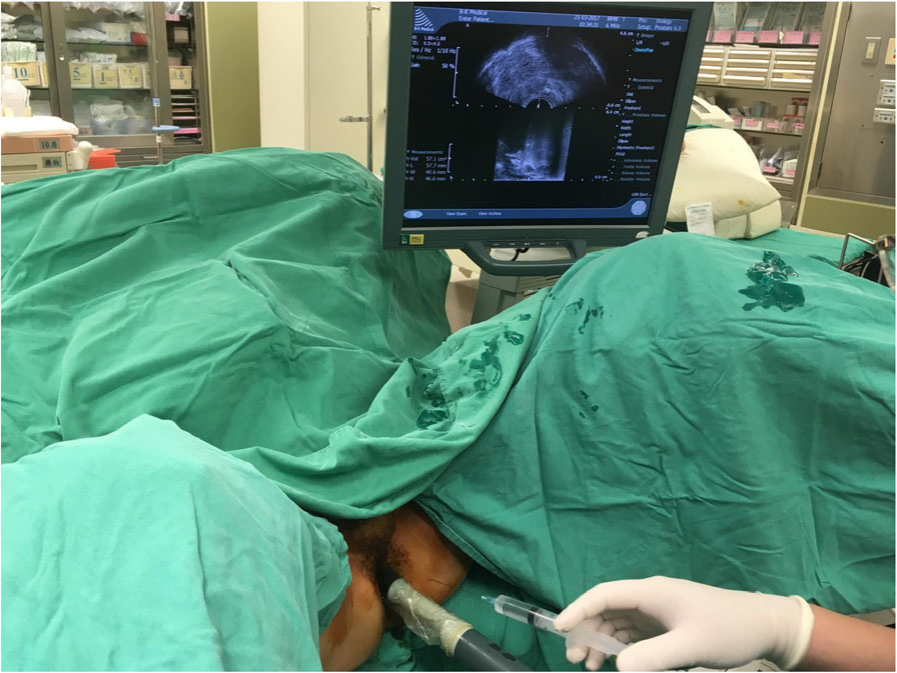
Transperineal prostate biopsy. Under axial and sagittal view of ultrasonography, 2% lidocaine was infiltrated through the 21-gauge fine needle from the perineum skin to prostate capsule

**Fig. 3:**
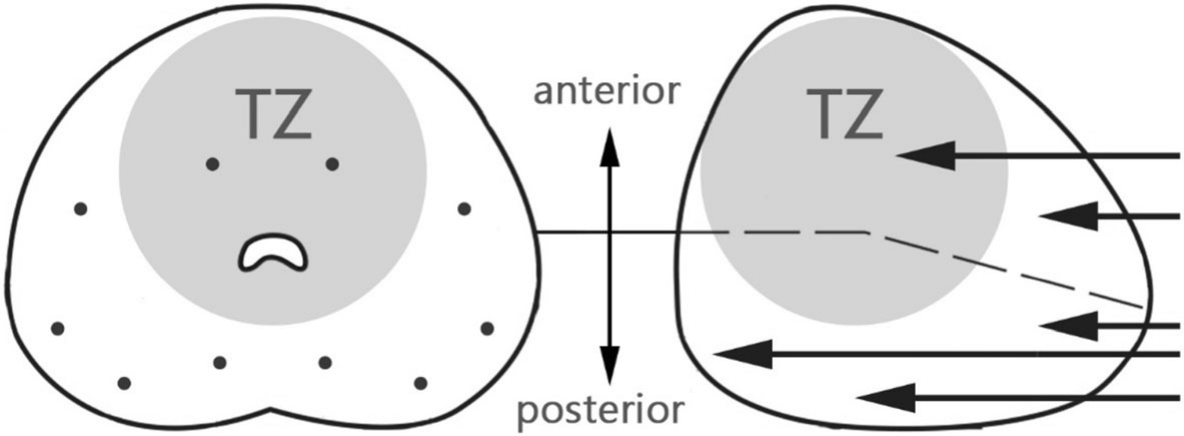
Transperineal prostate biopsy mapping. Systemic biopsy includes 6 cores over the peripheral zone, 2 cores over the anterior-apical area and 2 cores over the transitional zone. Biopsy core numbers may vary according to prostate volume and additional lesions

For transperineal (TP) prostate biopsy: The patient was placed in the lithotomy position with well-disinfected perineum preparation. Under the transrectal ultrasonography guidance (bk3000; BK Medical Aps, Copenhagen, Denmark), the Biplane prostate probe (Prostate Biplane E14C4t/4 M Hz; BK Medical Aps) was introduced for localization (Fig. 2). Systemic transperineal 18-gauge needle was inserted with 0–2 cores on the anterior-apical region, 2 cores on the transitional zone, and 6–8 cores on the peripheral zone (Fig. 3). Perioperative antibiotic treatment wasn’t needed during the procedure.

**Fig. 4:**
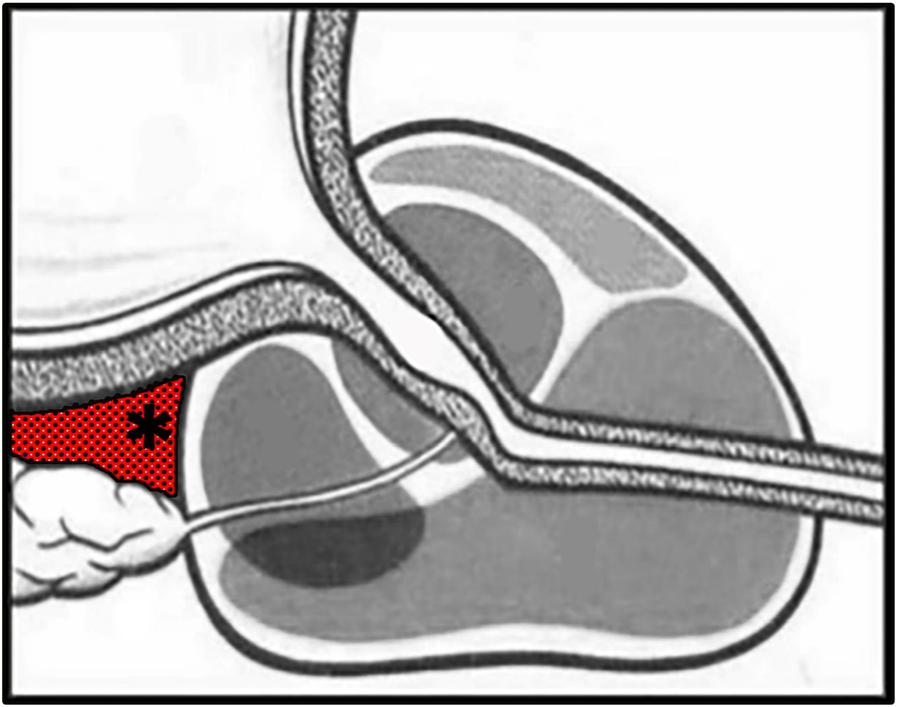
Periprostatic nerve block. Under transrectal ultrasound guidance, 2% lidocaine was injected into the triangular asterisk area where prostate neurovascular bundle passes through

**Fig. 5:**
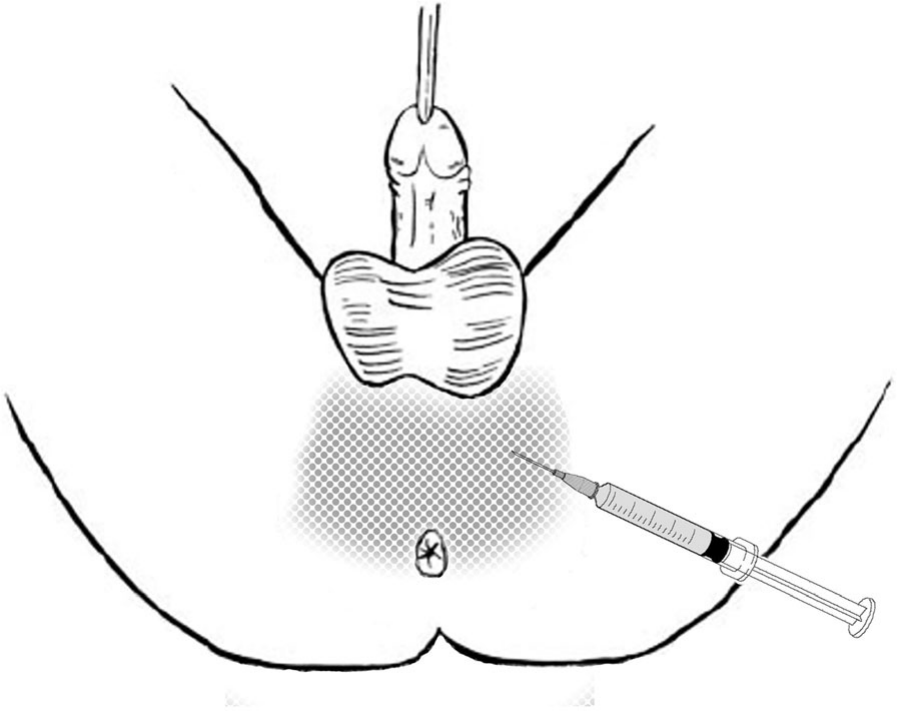
Transperineal biopsy under local anesthesia. 2% lidocaine was adequately infiltrated over the perineum for multiple biopsy puncture through skin

Anesthesia procedure: Patients of the TR group were treated with a lidocaine cream lubricant through the transrectal ultrasound puncturing hole. In the TP group, the patients underwent local anesthesia or intravenous general anesthesia. Local anesthesia with a peri-prostate nerve block effect (TP: LA + PPNB) was conducted with a 21-gauge needle. 2% 10 ml lidocaine was injected into the bilateral prostate basal area where the major neurovascular bundle transverses through [8, 9] (Fig. 4). Besides, additional 10 ml lidocaine was injected around the perineum skin for local anesthesia (Fig. 5).

### Data collection and analysis

All the procedures were carried out via the OPD surgery without admission by attending physicians (PHC, CSK, and WCL). Cancer detection rates and complications were independently collected and analyzed by another team member of this research (GLH).

### Complication definition

Urine retention is defined as being hard to urinate with catheterization within the 7 days after biopsy. Epididymitis was diagnosed by the physical examination and testis ultrasonography. Post-procedure sepsis was defined as fever > 38.5 °C with single or multiple organ dysfunctions during hospital admission. Hematuria and perineal hematoma were collected by patient-reported results in 7 days after the bioptic procedure.

### Pain score evaluation

An independent nurse in the operation room recorded the VAS pain scores immediately after the procedure. All patients were asked about their willingness to undergo re-biopsy if false negative findings were reported.

### Statistics and analysis

IBM SPSS Statistics ver. 21.0 (SPSS Inc., Chicago, IL) was used for data analysis with Student *t*-test and Mann-Whitney U test on the basic characteristic data of the two groups. Binary outcomes of the infection-related complications were analyzed by Chi-square test. Analysis of VAS pain scores under TR and TP was performed using the Kruskal-Wallis test.

## Results

As shown in Table 1, the mean age of the patients in TP and TR groups was 66.6 and 67.1 years old respectively. The median PSA value for the males in TP and TR groups was 9.3 and 10.9 ng/ml, respectively. There were no statistical differences when comparing the age, PSA values, DRE positivity, and median biopsy core number of the TP group with those of the TR group (Table 1). Nevertheless, the median prostate volume of the TR patient group was significantly greater than the of the TP group (Median, 35 ml vs 32.5 ml, *p* = 0.015).

**Table 1:** Characteristics in the trans-perineal (TP) and trans-rectal prostate (TR) biopsy

	TP(*n* = 130)	TR(*n* = 108)	*P* value
Age (years)	66.6 ± 8.81	67.1 ± 8.45	0.708^b^
Median PSA level (ng/ml) [25–75%]	9.3[6.3–20.3]	10.9[7.8–17]	0.099^a^
PSA < 4 ng/ml	3/130	7/108	0.11^c^
10 > PSA > 4 ng/ml	62/130	52/108	0.944^c^
PSA > 10 ng/ml	66/130	49/108	0.407^c^
DRE positive	49/130	50/108	0.18^c^
Median Biopsy core number [25–75%]	10[10–10]	10[10–10]	0.574^a^
Prostate Volume [25–75%]	32.5[27–41]	35[29–47]	0.015^a^
Prophylaxis abx use	0/130	Baktar: 28/108 Ceftibuten:25/108 Ciprofloxacin:5/108 Levofloxacin:50/108	<0.001^c^
Anesthesia procedure	IVG: 32/130 LA + PPNB: 98/130	Lidocaine Jelly lubrication	

*TP* trans-perineal prostate biopsy, *TR* trans-rectal prostate biopsy, *PSA* prostate specific antigen, *DRE* digital rectal examination, ^a^: Mann-Whitney test. ^b^: Student T test. ^c^: Chi-Square test

45% of the patients in the TP group (*n* = 130) and 49% of those in the TR group (*n* = 108) were separately diagnosed with prostate cancer (Table 2). The cancer detection rates, with respect to the two groups, were statistically comparable (*p* = 0.492). Regarding the post-bioptic infectious complications, 0.9, 4.6, 12, and 6.4%, 6.4% of the patients in the TR group were shown to have the following conditions: epididymitis, prostatitis, UTI, fever (> 38.5 °C), and sepsis, respectively. In the TP group, though there were 2.2 and 0.7% of patients who were further diagnosed with UTI and prostatitis, no one else was shown to get epididymitis, fever, and sepsis following the bioptic procedure. Statistically, except for prostatitis and epididymitis, all infection-related complications were lower in the TP group. (Table 2.)

**Table 2:** Complications, cancer detection rate and VAS between the trans-perineal (TP) and trans-rectal prostate (TR) biopsy

	TP (*n* = 130)	TR (*n* = 108)	*P* value
Gross hematuria	7/130(5.3%)	15/108(13.8%)	0.024^#^
Urine retention	4/130(3%)	9/108(12.0%)	0.076^#^
Perineal hematoma	2/130(1.5%)	0/101	0.196^#^
Epididymitis	0/130	1/108(0.9%)	0.272^#^
Prostatitis	1/130(0.7%)	5/108(4.6%)	0.059^#^
UTI	3/130(2.2%)	13/108(12.0%)	0.003^#^
Fever > 38.5	0/130	7/108(6.4%)	0.003^#^
Sepsis	0/130	7/108(6.4%)	0.015^#^
Hospitalization for complication	0/130	8/108(7.4%)	0.008^#^
VAS [25–75%]	3[2–4]	3[3–4]	0.239^◎^
Cancer detection rate	58/130(45%)	53/108(49%)	0.492^#^

*UTI* urinary tract infection, *VAS* Visual analog score. ^#^: Chi-square test. ^◎^: Mann-Whitney test

**Fig. 6:**
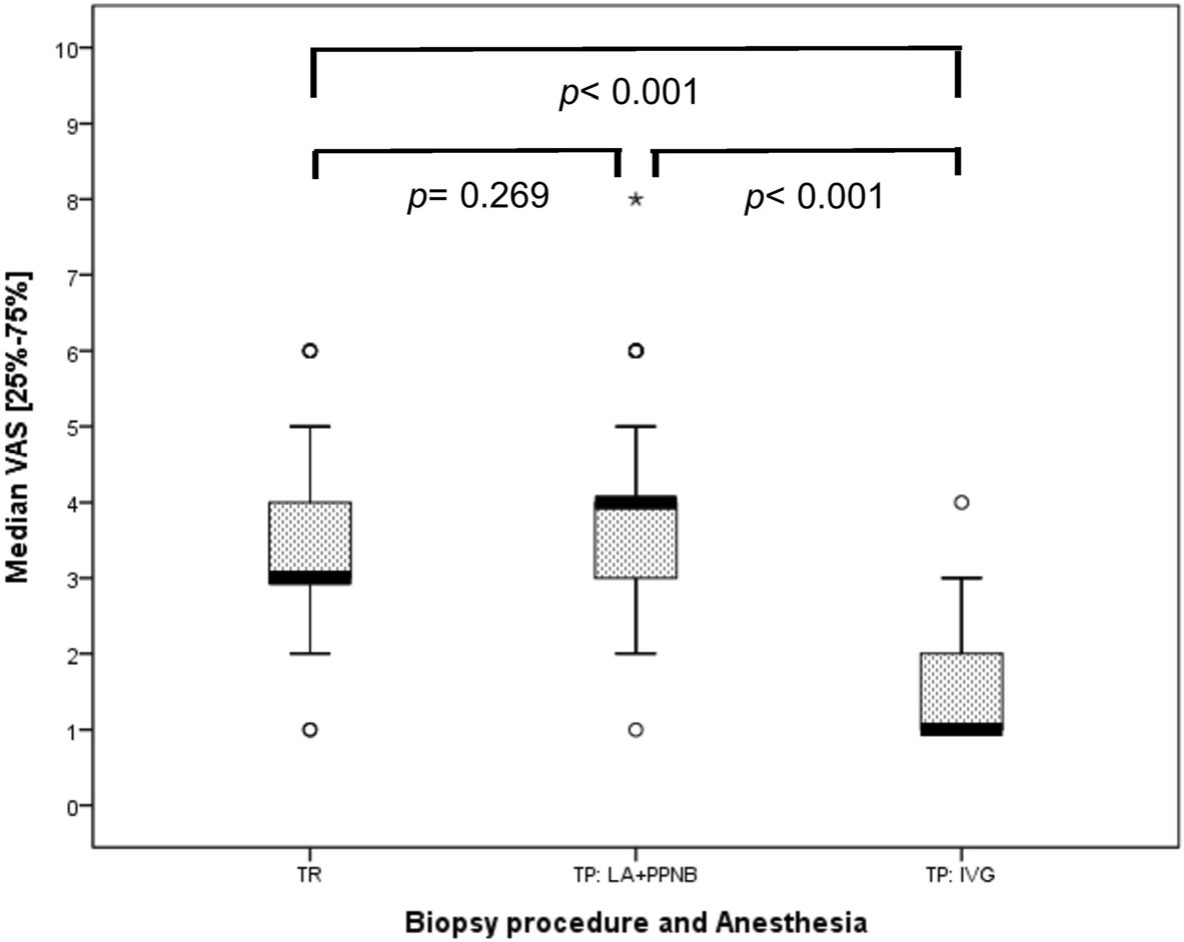
Median VAS scores and error bar between transperineal and transrectal procedures. TR: transrectal prostate biopsy. TP: LA + PPNB: transperineal approach, local anesthesia & peri-prostatic nerve block; IVG: Intravenous general anesthesia. Patients assigned to the TP group were subjected to anesthesia either locally via perineal injection or generally through intravenous injection

As for the minor complications, gross hematuria was found in 13.8 and 5.3% of the patients in TR and TP groups (*p* = 0.024), respectively (Table 2). The portion of the patients who had urine retention after the TR procedure was mildly higher, as compared to the TP procedure (12% v.s. 3%, *p* = 0.076). Merely two of the patients in the TP group had perineal hematomas after the bioptic procedure, whereas none of the patients in the TR group had such complication (*p* = 0.196) (Table 2). Taken together, these results suggest that patients undergoing transperineal prostate biopsy are at lower risk of post-bioptic complications. Therefore, the hospitalization rate of the patients in the TP group (0%) was obviously lower than that in the TR group (7.4%) (Table 2). In addition, median (25–75% quartiles) of VAS scores of the patients in the TP and TR groups was 3 [3, 4] and 4 [3–5], respectively, but there was no statistically significant difference between these two groups (*p = 0.085*) (Table 3) (Fig. 6).

**Table 3:** Mean VAS and error bar among the different biopsy procedures

	TR(*n* = 108)	TP:LA + PPNB(*n* = 98)
Median VAS [25–75%]	3[3–4]	4[3–5]
Independent Kruskal-Wallis test	*p* = 0.085	

*TR* transrectal prostate biopsy, TP: LA + PPNB: transperineal approach, local anesthesia with periprostatatic nerve block

## Discussion

Transrectal prostate biopsy has been accepted as the standard procedure for prostate cancer diagnosis and evolved into an extended prostate biopsy that includes up to ten and even twelve cores over the past decades [10]. Transperineal biopsy was initially deemed as an alternative cancer detection tool after negative TR biopsy, while the subsequent study results showed non-inferiority of TP biopsy for prostate cancer detection when either of these two approaches were solely performed [7]. For the time being, passage of the biopsy needle through the rectal wall is a mainstream procedure for prostate diagnosis in the United States, while transperineal biopsy is comparatively more accepted in Europe and Japan. A prospective study carried out by Watanabe et al. showed that the extensive 12-core approach simultaneously combining TR and TP biopsies could significantly improve the overall cancer detection rate, and was particularly effective for males with a negative DRE finding, accompanied by PSA levels of 4–10 ng/ml [11]. As an attempt for initial prostate biopsy by the transperineal approach, Kojima et al. demonstrated that extended TP biopsy up to 10–12 cores could lead to a better cancer detection rate, as compared to 6-core TP biopsy [12]. In our study, the averagely 10-core prostate biopsy was performed in the patients assigned to the TP and TR groups, respectively. The cancer detection rates, with respect to TP biopsy and TR biopsy (45 and 49%), were statistically comparable. Which suggests TP biopsy as a primary diagnostic tool not inferior to TR biopsy. Correspondingly, TP biopsy is, in general, regarded as an alternative strategy when TR biopsy fails or the quantity of obtained samples isn’t sufficient [13, 14].

TR biopsy is widely used in the US, and the complications following prostate biopsy have been well documented [15]. Post-bioptic infectious complications and the ensuing mortality are nightmares to urologists serving in the areas where multi-resistant bacteria are increasing at an alarming rate [16, 17]. The incidence of infectious complications after TR biopsy is reported to range from 0.1–7% and the rate of hospital admission due to post-bioptic infection is 0.6–4.1% [5]. *Escherichia coli* is the most common pathogen with fluoroquinolone-resistance identified in patients with post-bioptic infectious complications. Risk factors for post-bioptic infection-related complications include: (1) previous use of fluoroquinolone, (2) diabetes mellitus (3) chronic obstructive pulmonary disease, (4) hospitalization in preceding months, and (5) long-term Foley catheter in-dwelling [17, 18]. Although pre-biopsy rectal swab and use of targeted prophylaxis antibiotics are advocated for high-risk patients, the evidence for the routine implication remains inadequate [19]. Changing the prophylaxis antibiotics from fluoroquinolone to third-generation cephalosporin or in combination with gentamicin or amikacin, however, may contribute to occurrence of ESBL-producing bacteria and eventually increase bacterial resistance [5]. Post-TR-biopsy sepsis is caused by direct inoculation of bacteria from the rectal mucosa into the prostate, blood vessels and urinary tract via the biopsy needle. The alternative for preventing the post-biopsy infectious complications is to use the TP biopsy gun that punctures through the disinfected perineal skin instead of the rectal mucosa. In our study, there were only 2.2 and 0.7% of the patients in the TP group who respectively diagnosed with post-bioptic UTI and prostatitis. In the TR group, up to 12% of the patients were diagnosed with post-bioptic UTI, while 6.4% of the patients went to the emergency room (ER) due to the post-bioptic fever and sepsis. Although all the sepsis patents made an uneventful recovery after treated with IV antibiotics, the rate of post-bioptic hospitalization for infectious complications was 7.4%, which resulted in unnecessary medical costs.

Grummet et al. described that only 5 cases (0.076%) of the 6609 patients undergoing TP biopsy were admitted to hospital for post-bioptic sepsis, and no infection related mortality occurred as a result [16]. In terms of TR prostate biopsy, incidence of post-bioptic bacteremia is up to 3.0~6.9% and may be up to one-quarter for the patients needing ICU monitoring and treatment [17, 20–24]. In this regard, performing prostate biopsy through the TP approach should be advocated as the cancer detection rate of TP biopsy is comparable to that of TR biopsy, and, more importantly, TP biopsy demonstrates lower risk of infection-related complications [25].

One of the most common complications after prostate biopsy is hematuria, with reported incidence of 2–84% depending on the definition, follow-up duration and methods [26–29]. In our study, the incidence of hematuria was 5.3 and 13.8% respectively for patients in the TP and TR groups (7/130 vs 15/108, *p* = 0.024). This significant difference in the rate of hematuria between the TP and TR groups might be attributable to the significant difference of the prostate volume between the two groups (35.4 ml vs 40.1 ml, *p* = 0.015). In our study, there were totally 22 hematuria patients, whose symptoms were self-limited, and all of them were subjected to the conservative treatment as per previous reports [26–28].

Risk factors for post prostate biopsy urine retention have been reported, including large prostate volume, bulging prostate transitional zone and high International Prostate Symptom Score (IPSS) scores [15, 30, 31]. In our study, the rate of urine retention for the patients in the TP and TR groups was 3 and 12%, respectively (*p* = 0.076). Larger prostate volume of the patients in the TR group may be suggested as the cause of higher urine retention rate found in these patients.

Perineal hematoma was identified in two of the patients in the TP group, possibly because no perineum compression took place after the procedure. On the contrary, none of the patients in the TR group had perineal hematoma, since the compression was done after the procedure. Pepe et al. reported, according to their experience of performing more than 4000 transperineal prostate biopsies, that the incidence of perineal hematoma was around 0.3–1% [32]. In our study, most hematoma was self-limited, and no more cases were found after routine compression of the TP puncture site after the 2 episodes.

Pain is of great concern when considering different biopsy protocols. According to previous studies [33–35], TR biopsy with lidocaine cream lubrication was worldwidely conducted in many hospitals with tolerability. Other alternative anesthetic procedures, like lidocaine spray administration or replacing lidocaine cream with EMLA cream were reported for a better control of the pain during TR biopsy as well [36, 37]. In contrast, use of the TP biopsy gun to penetrate through the sensitive perineum skin and neurovascular bundles surrounding the prostate capsule is painful and intolerable. General or spinal anesthesia may be required when the procedure is carried out without adequate pain control and sedation [38–40]. However, general anesthesia is not feasible for high anesthesia-risk patients or in anesthetist resource-limited areas. Periprostatic nerve block (PPNB) has been reported as an effective method for alleviating pain and discomfort during TR biopsy and transurethral surgery [41, 42]. Local lidocaine anesthesia with sufficient perineum skin infiltration and PPNB provides an alternative for those patients. In our study, under local anesthesia, there were no significant differences in VAS scores between the patients in the TR and TP groups (TR: 3 [3, 4], TP:LA + PPNB: 3 [3–5], *p* = 0.085). Based on the experience of performing 50 TP prostate biopsies under local anesthesia, Smith et al. reported that the mean VAS scores respectively for probe insertion, LA injection, and bioptic procedure were 3.28, 3.29, and 2.88, which suggests feasibility of TP under local anesthesia [43]. Our clinical observation also demonstrated the feasibility of TP prostate biopsy under local anesthesia. In contrast, Udeh et al. reported that a much higher level of experienced pain was detected in the patients undergoing anesthetic TP biopsy, as compared those receiving TR biopsy under local anesthesia [44]. In this respect, digital-guided biopsy should be replaced with ultrasound-guided biopsy for the TP procedure, and full lidocaine cream premedication is also recommended for decreasing the discomfort during the procedure [34]. Besides, finger manipulation may exacerbate the pain when performing the biopsy. Younger patient age, which denote the higher drug metabolism and more analgesics use, may also account for the sensitiveness of the patients to pain [45]. In our study, there were 15 cases of the patients in the TP group showing the VAS score greater than 5. In our experience, adequate local anesthesia injection into the perineum skin, together with sufficient infiltration time, can alleviate pain and discomfort caused multiple punctures through the skin, and its efficiency is as important as periprostatic nerve block.

With an increase of antibiotic-resistant bacteria in developing countries, TP biopsy procedures have gained particular interest in these regions. However, most clinical studies regarding TP biopsy were carried out under general and spinal anesthesia. In this study, we utilized the LA plus PPNB approach to enhance the pain control and tolerability in TP biopsy patients. Larger sample size and randomized-design studies will be considered for validation in our future study.

Limitations of this study include lack of randomization in a relatively small study population of 238 patient cases collected in a single institute. However, double-blind designs for TP and TR procedures remain practical when performing the operation and post-operation evaluation. This prospective preliminary result provides the realistic nature for transperineal prostate biopsy implication.

## Conclusions

The cancer detection rates were comparable to both transperineal (TP) and transrectal (TR) prostate bioptic approaches. However, the ratios of post-bioptic infectious complications in patients undergoing TP biopsy were much lower than those receiving conventional transrectal prostate biopsy. Besides, TP biopsy under local anesthesia is feasible for high general anesthesia-risk patients, particularly in the areas where the emergence of antibiotic-resistance is rising or the resources are limited.

## Data Availability

The datasets used and/or analysed during the current study are available from the corresponding author on reasonable request.

## Abbreviations

DRE: digital rectal exam
ESBL: extended-spectrum β-lactamase
IM: intramuscular
IPSS: International Prostate Symptom Score
LA: lidocaine anesthesia
OPD: outpatient department
PC: prostate cancer
PPNB: periprostatic nerve block
PSA: prostate-specific antigen
PZ: peripheral zone
TP: transperineal
TR: transrectal
TZ: transitional zone
UTI: urinary tract infection
VAS: visual analog scale
